# Positive Regulation by GABA_B_R1 Subunit of Leptin Expression through Gene Transactivation in Adipocytes

**DOI:** 10.1371/journal.pone.0020167

**Published:** 2011-05-31

**Authors:** Yukari Nakamura, Eiichi Hinoi, Takeshi Takarada, Yoshifumi Takahata, Tomomi Yamamoto, Hiroyuki Fujita, Saya Takada, Syota Hashizume, Yukio Yoneda

**Affiliations:** Laboratory of Molecular Pharmacology, Division of Pharmaceutical Sciences, Kanazawa University Graduate School of Natural Science and Technology, Kanazawa, Ishikawa, Japan; University of Houston, United States of America

## Abstract

**Background:**

The view that γ-aminobutyric acid (GABA) plays a functional role in non-neuronal tissues, in addition to an inhibitory neurotransmitter role in the mammalian central nervous system, is prevailing, while little attention has been paid to GABAergic signaling machineries expressed by adipocytes to date. In this study, we attempted to demonstrate the possible functional expression of GABAergic signaling machineries by adipocytes.

**Methodology/Principal Findings:**

GABA_B_ receptor 1 (GABA_B_R1) subunit was constitutively expressed by mouse embryonic fibroblasts differentiated into adipocytes and adipocytic 3T3-L1 cells in culture, as well as mouse white adipose tissue, with no responsiveness to GABA_B_R ligands. However, no prominent expression was seen with mRNA for GABA_B_R2 subunit required for heteromeric orchestration of the functional GABA_B_R by any adipocytic cells and tissues. Leptin mRNA expression was significantly and selectively decreased in adipose tissue and embryonic fibroblasts, along with drastically reduced plasma leptin levels, in GABA_B_R1-null mice than in wild-type mice. Knockdown by siRNA of GABA_B_R1 subunit led to significant decreases in leptin promoter activity and leptin mRNA levels in 3T3-L1 cells.

**Conclusions/Significance:**

Our results indicate that GABA_B_R1 subunit is constitutively expressed by adipocytes to primarily regulate leptin expression at the transcriptional level through a mechanism not relevant to the function as a partner of heterodimeric assembly to the functional GABA_B_R.

## Introduction

γ-Aminobutyric acid (GABA) is known as one of the most abundant inhibitory amino acid neurotransmitters in the mammalian central nervous system (CNS) with three different signal receptor subtypes, including GABA_A_ receptor (GABA_A_R), GABA_B_ receptor (GABA_B_R) and GABA_C_ receptor (GABA_C_R), to date [1]. The ionotropic GABA_A_R and GABA_C_R subtypes are responsible for the rapid component of inhibitory postsynaptic potentials through activation of ion channels permeable to chloride ions. The GABA_A_R is a heteromeric protein complex composed of a number of different subunits, while the GABA_C_R is derived from the assembly between various isoforms of the rho subunit [2]. By contrast, the metabotropic GABA_B_R subtype belongs to a superfamily of the seven-transmembrane-domain receptors with high similarity to metabotropic receptors for the excitatory amino acid neurotransmitter L-glutamate. The GABA_B_R couples to adenylyl cyclase through trimeric G-proteins to inhibit intracellular cAMP formation, in addition to negative and positive modulation of the activities of voltage-sensitive Ca^2+^ and K^+^ channels, respectively [3].

In addition to these GABA receptors required for signal input, GABA participates in inhibitory neurotransmission through the mechanisms relevant to different signaling machineries located in GABAergic synapses. These include L-glutamic acid decarboxylase (GAD) for GABA synthesis [4], vesicular GABA transporter (VGAT) for the condensation in synaptic vesicles for subsequent exocytotic release into synaptic cleft [5] and high-affinity GABA transporter (GAT) for the clearance from synaptic cleft into adjacent glia and neurons [6]. These GABAergic signaling machineries are found in a variety of non-neuronal and peripheral tissues such as bone, heart, lung, kidney, adrenal, pancreas, liver, spleen and uterus outside the CNS [7]–[9]. Both GABA [10] and GAD [11] are highly condensed in β-cells of Langerhans islets, for example, while GABA is released from β-cells to exert a paracrine inhibitory effect on glucagon secretion from neighboring α-cells through activating GABA_A_R [12], as well as an autocrine suppressive effect on insulin secretion through activation of GABA_B_R [13], in pancreas. Accordingly, GABAergic signaling machineries could regulate energy balance through autocrine and/or paracrine mechanisms in these peripheral tissues *in vivo*. However, little attention has been paid to GABAergic signaling machineries expressed by adipocytes, which undoubtedly play a pivotal role in the regulation of body energy homeostasis, to date.

In the present study, therefore, we have attempted to demonstrate the functional expression of GABAergic signaling machineries by adipocytes in order to clarify the physiological and pathological significance of GABAergic signals in energy metabolism *in vivo* using both white adipose tissue (WAT) and embryonic fibroblast (EF) endowed to differentiate into adipocyte from mice defective of the GABA_B_R orchestration partner GABA_B_R1 subunit, in addition to murine pre-adipocytic 3T3-L1 cells in culture.

## Materials and Methods

### Materials

Pre-adipocytic 3T3-L1 cells were purchased from ATCC (Manassas, VA, USA). Std-ddY mice were supplied by SANKYO LABO SERVICE (Toyama, Japan). Bovine insulin, 3-isobutyl-1-methylxanthine (IBMX), 2-hydroxysaclofen and anti β-tubulin antibody were purchased from Sigma Chemicals (St. Louis, MO, USA). ECL™ detection reagent was obtained from Amersham Biosciences (Piscataway, NJ, USA). Alpha minimal essential medium (αMEM), Dulbecco's Modified Eagle Medium (DMEM), Opti-MEM1 Reduced-serum Medium and ethidium bromide were obtained from Gibco BRL (Grand Island, NY, USA). Baclofen, saclofen and CGP46381 were purchased from TOCRIS (Ballwin, MO, USA). Taq polymerase was obtained from Takara (Tokyo, Japan). Dual luciferase assay system and pRL-CMV were purchased from Promega (Madison, WI, USA). M-MLV Reverse Transcriptase, Plus reagent, LipofectamineRNAiMAX, Lipofectamin2000, Stealth™ RNAi Negative Control and GABA_B_R1 RNAi were supplied by Invitrogen Corp. (Carlsbad, CA, USA). A pGL3-PGC1α-Luc containing 5′ flanking sequence of mouse peroxisome proliferator-activated receptor gamma coactivator 1-α (PGC1α) gene (−2533 to +78) was kindly donated by B. Spiegelman (Harvard Medical School, Boston, MA, USA) [14]. A pCRE-Luc was provided by STRATAGENE (La Jolla, CA, USA). Anti-GABA_B_R1 antibody, anti-rabbit IgG and anti-mouse IgG were purchased from SANTA CRUZ (Santa Cruz, CA, USA). ISOGEN and dexamethasone (DEX) were obtained from WAKO (Osaka, Japan). Other chemicals used were all of the highest purity commercially available.

### Animals

The BALB/c GABA_B_R1-null mice were kindly provided by Dr. K. Kaupmann (Novartis International AG, Basel, Switzerland). Wild-type (WT) and GABA_B_R1-null mice were obtained from heterozygous breeding in a university animal facility at room temperature, in a 12 h light/dark cycle with lights on at 8:45 a.m. and food and water *ad libitum*. Genotyping was done with reverse transcription polymerase chain reaction (RT-PCR) analysis using specific primers as described previously [15]. Epididymal visceral WAT and interscapular brown adipose tissue (BAT) were individually removed from animals after decapitation, followed by rinsing with phosphate-buffered saline (PBS) and subsequent measurement of wet weight. The protocol was reviewed and approved by the Institutional Review Board of Kanazawa University (AP-101806) with an effort to minimize the number of animals used and their suffering.

### Culture of 3T3-L1 cells

The murine pre-adipocytic cell line 3T3-L1 cells were plated at a density of 2.5×10^4^ cells/cm^2^ in DMEM containing 10% fetal bovine serum (FBS) and then cultured at 37°C until confluence under 5% CO_2_, followed by medium change to DMEM with 2.5 µg/ml insulin, 0.5 µM DEX and 0.5 mM IBMX for subsequent culture for an additional 48 h. Culture medium was then changed to DMEM containing 2.5 µg/ml insulin alone, followed by further culture for different periods up to 16 days. Medium was changed every three days. Exposure to baclofen and saclofen was sustainably during cell culture at 37°C under 5% CO_2_.

### Preparation of primary EF and induction of adipogenic differentiation

The foster mothers were sacrificed at 13.5 to 14.5 days past coitus, the embryos were dissected from the uterus, and the extra-embryonic membranes, head and viscera were removed. The embryos were cut into small pieces with scissors and soaked for 30 min in 4 ml of 0.25% trypsin-EDTA at room temperature with shaking, and then inactivated with αMEM containing 10% FBS. Cells were suspended by pipetting, plated on five 10-cm dishes per embryo, and incubated at 37°C for 48 h under 5% CO_2_. After 48 h, adherent cells were trypsinized, counted, and replated at a density of 2.5×10^4^ cells/cm^2^ in αMEM with 10% FBS to induce adipocyte differentiation. Cells were cultured until confluence, followed by medium change to αMEM with 2.5 µg/ml insulin, 0.5 µM DEX, 0.5 mM IBMX and 10% FBS for subsequent culture for 48 h. Culture medium was then changed to αMEM containing 2.5 µg/ml insulin and 10% FBS, followed by further culture for different periods up to 16 days. Medium was changed every other day. For overexpression of GABA_B_R1 subunit, moreover, secondary EF prepared from GABA_B_R1-null or WT mice was transiently transfected with 2.5 µg pCI-GABA_B_R1 or pCI empty vector (EV) using Lipofectamine LTX/Plus reagent for 24 h, followed by further culture with adipogenic differentiation inducers described above for an additional 2 days and subsequent determination of leptin mRNA levels with a real time-based RT-PCR.

### RT-PCR analysis

Cultured cells were superficially washed with PBS twice, followed by extraction of total RNA according to the standard ISOGEN procedure and then subjected to the synthesis of cDNA as described previously [16]. Epididymal WAT was homogenized in ISOGEN, followed by centrifugation at 4°C for 15 min at 20,000 g and subsequent collection of lower layer without fat layer for extraction of total RNA. The individual cDNA species were amplified in a reaction mixture containing a cDNA aliquot, PCR buffer, dNTPs, the relevant sense and antisense primers (Table 1) and rTaq DNA polymerase. Reactions were initiated by incubating at 94°C for 5 min and PCR (denaturation at 94°C for 1 min, annealing at appropriate temperature for 1 min and extension at 72°C for 1 min) was performed with a final extension at 72°C for 10 min. The PCR product was detected as a single band at the corresponding position expected. Quantitative analysis was done at the cycle number with high linearity between mRNA expression and cDNA production using primers for the housekeeping gene glyceraldehyde-3-phosphate dehydrogenase (GAPDH) as described previously [17]. Electrophoresis was run for an aliquot of PCR amplification products on a 1.5% agarose gel, followed by detection of DNA with ethidium bromide. Densitometry was done with the individual PCR products by using a densitograph, followed by calculation of ratios of expression of mRNA for each gene over that for GAPDH [18]. Alternatively, mRNA expression was quantified by real time-based RT-PCR using a MiniOpticon™ with an iQ SYBR Green Supermix for each primer set (Table 2). The relative amount of each transcript was normalized by the expression of GAPDH in a real time-based RT-PCR.

**Table 1:** Primers used for RT-PCR.

Genes	Upstream (5′- 3′)	Downstream (5′- 3′)	Estimated base pair
GABA_A_R α1	CATTCTGAGCACTCTCTCGGGAAG	GTGATACGCAGGAGTTTATTGGGC	396
α2	AGGTTGGTGCTGGCTAACATCCAA	AACGGAGTCAGAAGCATTGTAAGTCC	549
α3	CCAGCAGCCCCAACCAAGAA	GTTTGCGGATCATGCCCTTG	325
α4	AAAACCTCCTCCAGAAGTTCCA	ATGTTAAATGCCCCAAATGTGACT	532
α5	TGACCCAAACCCTCCTTGTCTTCT	ACCGCAGCCTTTCATCTTTCC	300
α6	TGTTGCTTCTCCCCTGGCTCTTC	TCTCATCGGACAGTCAGCGTTG	473
β1	GTTTGGGGCTTCTCTCTTTTCCT	GTTTGGGGCTTCTCTCTTTTCCT	578
β2	CAGGTTCTTATCCCAGATTGTCCC	GGTCCATCTTGTTGACATCCAGG	408
β3	CTTTTCGGCATCTTCTCGGC	CCACGCCAGTAACAGCCTTG	587
γ1	TAGTAACAATAAAGGAAAAACCACCAGA	CCAGATTGAACAAGGCAAAAGCT	296
γ2	TGGTGACTATGTGGTTATGTCCGTG	AGGTGGGTGGCATTGTTCATTT	423
γ3	TTCTCCTCTGCCTGTTCTCGG	CGAAGGCGACTGTCTGTCCAG	304
δ	GACTACGTGGGCTCCAACCTGGA	ACTGTGGAGGTGATGCGGATGCT	398
GABA_B_R 1a,1b	GTGACCATGATCCTTTCCAG	CAACAGTCGGGACTTCTCTT	210
2	CTGAGGACAAACCCTGCGC	GATGTCTTCTATGGGGTC	408
GABA_C_R ρ1, ρ2	ATGTGCAACATGGACTTCAGC	TGATGGTGGACATGGTCAGCA	394
ρ3	TGTCCACAATCGTCACTGGT	CCTCCCAGGGATTTTCTCT	362
GAD65	ACGCTTCGACCTCCCTCATGTCCTGT	GAATCAGACAGCTTTCGGAGTTGG	698
GAD67	CCGGGTGTTCCGTAAAGAAGAACCG	GTGTCGCTGGAGAGTCAGCTCTGC	541
GAT1	CACGCTCCTGGCTTTTAGTC	CCAGAACAGAGTGGCAGTGA	552
GAT2	GTCCACAGGCAAGGTTGTT	ACTCTTTCGGAGCTGCTGG	556
GAT3	GAATTCCAGAAGGCCAATGA	AAGCCCAGGATGGAGAAGAT	494
GAT4	TGCTGAGGTGGCAGAATCAGGTCC	TGTTGTACTTGAGCGGTTTG	480
VGAT	CCTGGGGTTGTTCCTCATCA	ACTTCCTTGGTCTCGTCGGC	649
GAPDH	ACCACAGTCCATGCCATCAC	TCCACCACCCTGTTGCTGTA	452
PPARγ	TATGGAGTTCATGCTTGTGA	CGGGAAGGACTTTATGTATG	315
C/EBPα	AAGGCCAAGAAGTCGGTGGA	CAGTTCACGGCTCAGCTGTT	186
LPL	GCCCAGCAACATTATCCAGT	AGCCCTTTCTCAAAGGCTTC	554
Fabp4	GAATGTGTTATGAAAGGCGTGA	ATAACCATATCCAATAAAATGCATC	195
β3-AR	CCTAGCTGTCACCAACCCTTT	GACGAAGAGCATCACAAGGAG	262
Adiponectin	GTTGCAAGCTCTCCTGTTCC	TGAAGAGGCTCACCTTCACA	530
TNF-α	ACGGCATGGATCTCAAAGAC	CGGACTCCGCAAAGTCTAAG	324

**Table 2:** Primers used for real time based PCR.

Genes	Upstream (5′- 3′)	Downstream (5′- 3′)
HSL	TATGGAGTTCATGCTTGTGA	CGGGAAGGACTTTATGTATG
Perilipin	AAGGCCAAGAAGTCGGTGGA	CAGTTCACGGCTCAGCTGTT
Leptin	GCCCAGCAACATTATCCAGT	AGCCCTTTCTCAAAGGCTTC
Resistin	GAATGTGTTATGAAAGGCGTGA	ATAACCATATCCAATAAAATGCATC
PGC1α	GTCAACAGCAAAAGCCACAA	TCTGGGGTCAGAGGAAGAGA
UCP1	TACCAAGCTGTGCGATGTCCA	GCACACAAACATGATGACGTTCC
GABA_B_R1	ACGTCACCTCGGAAGGTTG	CACAGGCAGGAAATTGATGGC
GAPDH	CGTCCCGTAGACAAAATGGT	TCGTTGATGGCAACAATCTC

### Immunoblotting analysis

Cultured cells were superficially washed with PBS twice, followed by homogenization in 10 mM HEPES-NaOH (pH 7.9) containing 10 mM KCl, 1 mM EDTA, 1 mM EGTA, 5 mM DTT, 10 mM NaF, 10 mM β-glycerophosphate and protease inhibitors. Homogenates were dissolved in 10 mM Tris-HCl buffer containing 2% sodium dodecylsulfate (SDS) and 5% 2-mercaptoethanol, followed by boiling for 10 min and subsequent loading of an aliquot for electrophoresis on a 7.5% SDS-polyacrylamide gel toward blotting to a polyvinylidene fluoride membrane. After blocking by 5% skim milk dissolved in 20 mM Tris-HCl buffer (pH 7.5) containing 137 mM NaCl and 0.1% Tween 20 (TBST), the membrane was incubated with one of antibodies against GABA_B_R1 and β-tubulin adequately diluted with TBST containing 1% skim milk, and then with the secondary antibody conjugated with horseradish peroxidase. Finally, the membrane was incubated with ECL™ detection reagent to detect immunoreactive proteins, followed by exposure to X-ray films for different periods to obtain films appropriate for subsequent quantitative densitometry.

### Oil red O stain

Cells were stained with Oil Red O under the standard procedures as described elsewhere [19]. In brief, cells were rinsed with PBS twice and fixed with 4% paraformaldehyde for 15 min. After successive washing twice with water and once with 30% and 60% isopropanol, cells were stained for 20 min with 0.3% Oil Red O dissolved in 60% isopropanol. Cells were then washed three times with 60% isopropanol and twice with 30% isopropanol for observation under an Olympus IMT-2-21 dissecting microscope. Cells stained were dissolved in 100% isopropanol for 10 min for subsequent quantification of the absorbance at 490 nm.

### Transient transfection for luciferase assay

Leptin promoter fragments at −1995/+125 bp were isolated from mouse genome DNA by PCR using the following primers: 5′- acgctagcCTGCAGCCATGGTTACCTC-3′ as an upstream primer containing NheI site and 5′- gcaagcttGACCTCCTTCTTGCCTCAG -3′ as a downstream primer containing HundIII. The PCR-amplified DNA products were cloned into the pGL3-basic. 3T3-L1 cells were seeded at 1.3×10^4^ cells/cm^2^ at 24-well plates and maintained at 37°C in a humidified 5% CO_2_ incubator. Transfection of 0.4 µg DNA with an internal control vector pRL-CMV to cells was performed by the Lipofection method using Lipofectamine LTX/Plus reagent for 24 h after cell seeding. Medium was replaced with DMEM containing 10% FBS after transfection, followed by exposure to a test drug for 24 h. Firefly and renilla luciferase activities were determined using the Dual Luciferase Assay system. 3T3-L1 cells were seeded at 2.5×10^4^ cells/cm^2^ in 24-well plates with siRNA and DNA complex using Lipofectamine 2000 according to the manufacturer's instructions for the reverse method. Medium was replaced with DMEM containing 10% FBS 24 h after transfection, followed by measurement of luciferase activity 48 h later.

### RNA interference

3T3-L1 cells were transfected with siRNA using LipofectamineRNAiMAX under the reverse transfection protocol according to the manufacturer's specification. Cells were then cultured in DMEM containing 10% FBS for 72 h after transfection, followed by either quantitative determination of GABA_B_R1 protein on immunoblotting analysis or induction of adipocyte differentiation for 48 h according to the aforementioned procedures. Total RNA was extracted for RT-PCR from 3T3-L1 cells transfected with siRNA after inducing adipocyte differentiation.

### Detection of plasma leptin and insulin levels

Blood was collected from the heart of WT and GABA_B_R1-null male mice at 4 weeks old under ether anesthesia using needles and syringe, followed by leaving on ice for 5 min and subsequent centrifugation at 4°C for 5 min at 20,000 g for collection of plasma. Plasma leptin was measured using Mouse and Rat Leptin ELISA kit (BioVendor R&D, Czech) according to manufacturer's protocols. Plasma insulin was measured using Mouse Insulin ELISA kit (Mercodia, Sweden).

### Measurement of food intake

Male WT and GABA_B_R1-null mice at 3 weeks old were weaned from the breast and singly housed for a week before food intake measurement. Mice were fed with powder diets for 1 week to measure the accumulated amount of food intake.

### Measurement of locomotor activity

Open-field test was performed for measurement of locomotor activity. The open-field arena was made of a white acrylic floor and wood walls (50×50× 45 cm arena, divided to 5×5 squares, each 10×10 cm), positioned on the floor in an air conditioned (25°C) room with no windows. Four weeks old male mice were individually placed in the center of the arena, and allowed to freely explore it. The test arena was thoroughly cleaned with 70% ethanol before introduction of new mice. The parameters observed were: ambulation or crossing (the number of squares crossed with all 4 paws) and number of rearing, both recorded for the last 10 min of the 12 min testing period.

### Basal blood glucose titers and glucose tolerance tests

Blood glucose was measured by ACCU-CHECK® Active glucose meter (Roche Diagnostics Japan) in tail blood. Glucose was intraperitoneally injected at 2 g/kg to 4-weeks-old WT and GABA_B_R1-null male mice fasted overnight for 15 to 16 h, followed by measurement at 0, 15, 30, 60, and 120 min after injection.

### Data analysis

Results are all expressed as the mean ± S.E. and the statistical significance was determined by the two-tailed and unpaired Students' *t*-test or the one-way analysis of variance ANOVA with Bonferroni/Dunnett post hoc test.

## Results

### Expression profiles of GABAergic signaling machineries

We first examined the expression profile of a variety of adipocyte differentiation markers in 3T3-L1 cells cultured with differentiation media including insulin, DEX and IBMX. mRNA expression was drastically increased for all adipocyte marker genes examined in proportion to culture periods until 8 days. These include fatty acid binding protein 4 (Fabp4), peroxisome proliferator-activated receptor-γ (PPARγ), lipoprotein lipase (LPL) and adiponectin (data not shown).

**Figure 1:**
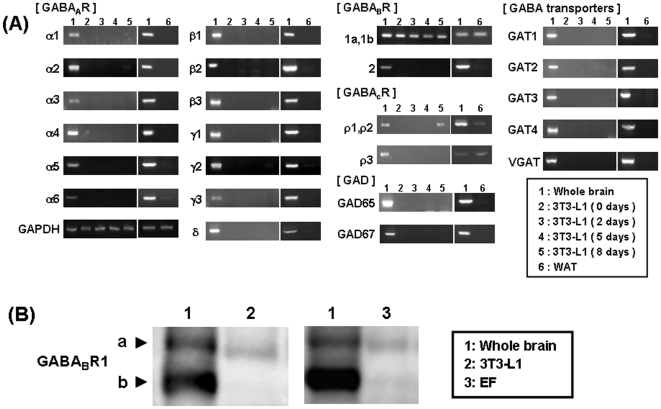
Expression profiles of GABAergic signaling molecules. (A) Total RNA was extracted from 3T3-L1 cells cultured for 2 to 8 days and epididymal WAT of male mice, followed by RT-PCR using specific primers for each molecule. (B) Both 3T3-L1 cells and EF were cultured until confluence, followed by detection of GABA_B_R1 subunit protein on immunoblotting analysis. Mouse whole brain was used as a positive control. Expression of mRNA and corresponding protein for GABA_B_R1 subunit was constitutively seen in adipocytic cells.

Under these conditions, mRNA expression for GABAergic signaling machineries was analyzed in 3T3-L1 cells. As shown in Figure 1A, mRNA expression was not seen for GABA_A_α1-6, β1-3, γ1-3, and δ subunits of GABA_A_R, and ρ3 subunits of GABA_C_R in 3T3-L1 cells cultured for different periods up to 8 days, with marked expression of mRNA for all GABA_A_R subunits and GABA_C_R subunits in mouse whole brain. Although constitutive expression was also seen with mRNA for GABA_B_R-1a and -1b isoforms, in addition to GABA_B_R2 subunit, in mouse whole brain, mRNA was constitutively detected for both GABA_B_R-1a and -1b subunits, but not for GABA_B_R2 subunit, in 3T3-L1 cells. No mRNA expression was seen for GAD, GAT and VGAT in 3T3-L1 cells cultured for different days examined. Sequencing analysis on amplified PCR products clearly confirmed the expression of mRNA for the corresponding GABA_B_R-1a and -1b subunits in 3T3-L1 cells (data not shown). In addition, mRNA expression for GABAergic signaling machineries was analyzed in WAT isolated from ddY strain mice. In accordance with expression profiles in 3T3-L1 cells, mRNA expression was seen for both GABA_B_R-1a and -1b subunits, but not for GABA_B_R2 subunit, in WAT (Figure 1A). Mouse whole brain, 3T3-L1 cells and EF were homogenized in buffer containing protease inhibitors, followed by immunoblotting analysis. Immunoreactivities were found to GABA_B_R1a subunit at approximately 130 kDa and GABA_B_R1b subunit at approximately 100 kDa, respectively, in both differentiated 3T3-L1 cells and EF, with much less amounts than in whole brain (Figure 1B).

### Absence of functional GABA_B_R from adipocytes

**Figure 2:**
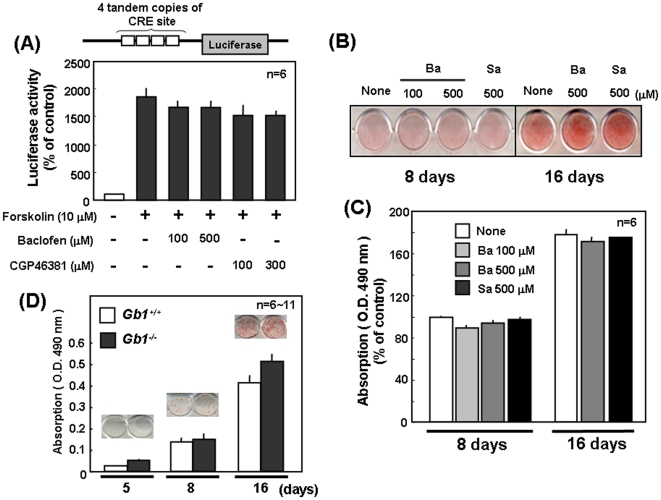
Lack of effects of GABA_B_R ligands on adipocytic differentiation. (A) 3T3-L1 cells were transfected with the luciferase vector containing 4 tandem copies of CRE site, followed by exposure to the GABA_B_R agonist baclofen, the GABA_B_R antagonist CPG46381 or forskolin for 24 h and subsequent determination of luciferase activity. 3T3-L1 cells were cultured for 8 to 16 days, in either the presence or absence of baclofen and the antagonist saclofen, followed by Oil red O staining. Typical micrographic pictures are shown in the panel (B), while in the panel (C) quantitative data are shown as the mean ± S.E in 5 independent determinations. (D) EF was isolated from WT and GABA_B_R1-null mice, followed by culture for 5 to 16 days and subsequent staining with Oil red O. Values are the mean ± S.E. from 6 to 11 different experiments. Neither agonist nor antagonist for GABA_B_R exhibited significant effects on adipogenesis. Ba, baclofen; Gb1, GABA_B_R1; Sa, saclofen.

In order to evaluate the possible function of GABA_B_R1 subunit expressed by adipocytes as metabotropic GABA_B_R, an attempt was made to determine whether GABA_B_R ligands affect luciferase activity in 3T3-L1 cells transfected with a cAMP responsive element (CRE) reporter plasmid. Exposure to forskolin resulted in a drastic increase in the luciferase activity in 3T3-L1 cells transfected with a CRE reporter plasmid, whereas the further addition of the GABA_B_R agonist baclofen or the GABA_B_R antagonist CGP46381 did not significantly affect the increase by forskolin (Figure 2A). Furthermore, 3T3-L1 cells were cultured for 8 to 16 days in either the presence or absence of the GABA_B_R agonist baclofen or the GABA_B_R antagonist saclofen, followed by determination of the lipid droplet accumulation with Oil red O staining. As shown in Figure 2B, lipid droplet accumulation was detected in 3T3-L1 cells cultured for 8 days with a marked increase at 16 days, while sustained exposure to either baclofen or saclofen failed to significantly affect the lipid droplet accumulation throughout the culture periods (Figure 2C). For further confirmation of the absence of functional GABA_B_R from adipocytic cells, EF was isolated from mice defective of GABA_B_R1 subunit, followed by culture in standard adipogenic induction medium to promote differentiate into adipocytes for subsequent Oil red O staining. As seen in 3T3-L1 cells, lipid droplet was drastically accumulated in EF cultured for 5 to 16 days irrespective of the knockout of GABA_B_R1 subunit, whereas no significant difference was detected in lipid droplet accumulation throughout the cultured periods up to 16 days between EF isolated from WT and GABA_B_R1-null mice (Figure 2D). Thus, GABA_B_R1 subunit alone would be constitutively expressed by adipocytic cells devoid of the partner GABA_B_R2 subunit required for functional GABA_B_R assembly.

### Expression profiles of adipocytic marker genes in GABA_B_R1-null mice

**Figure 3:**
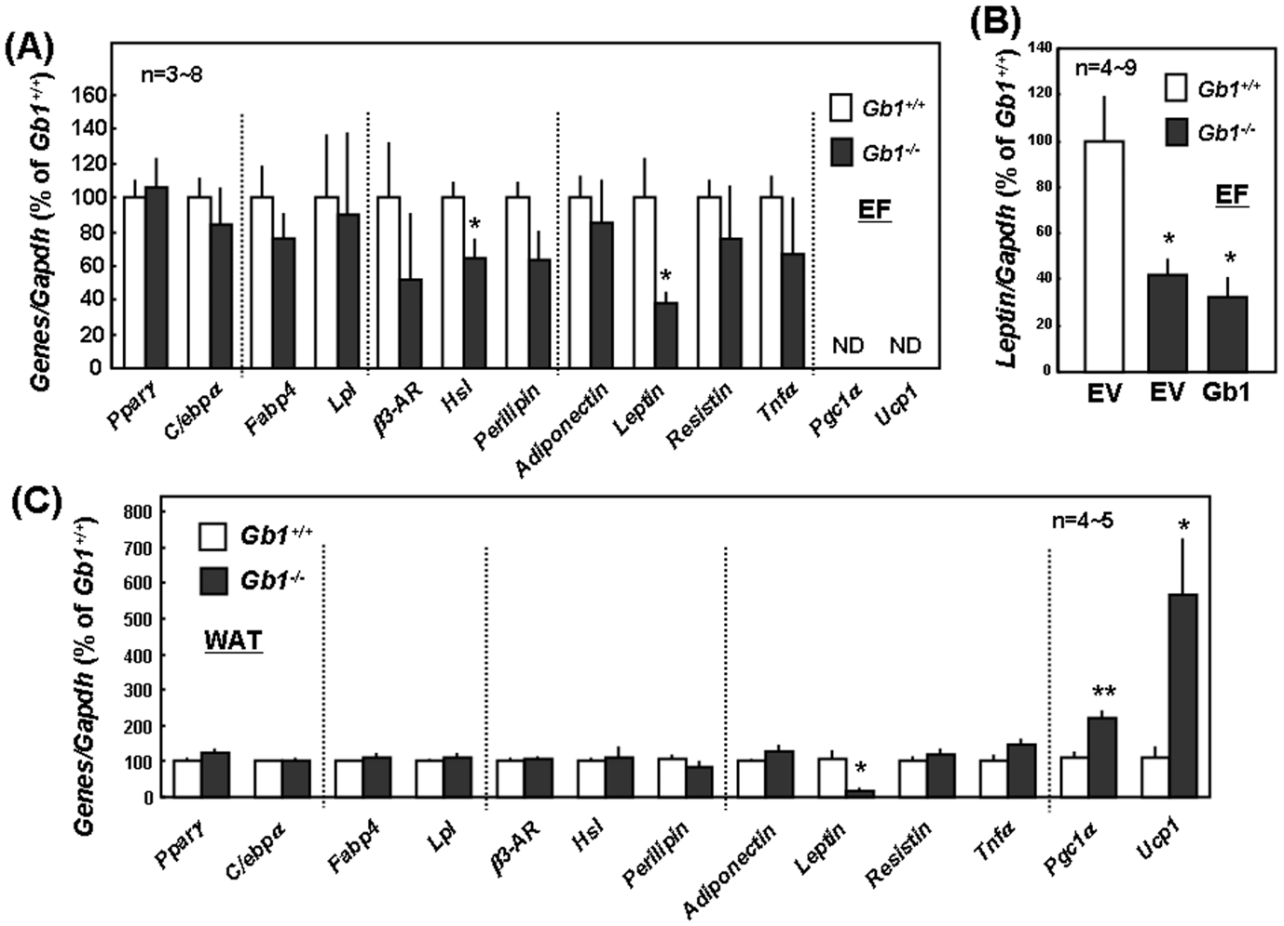
Expression profile of marker genes in adipocytes. (A) Total RNA was extracted from EF cultured for 2 days. (B) Total RNA was extracted from EF transfected with GABA_B_R1 expression vector. (C) Total RNA was extracted from epididymal WAT of male mice at 4 weeks old. Values are the mean ± S.E. from 3 to 8 different experiments. *P<0.05; **P<0.01, significantly different from each control value obtained in WT mice. Selective downregulation was seen in leptin mRNA expression in both cultured EF and WAT prepared from GABA_B_R1-null mice, while marked upregulation was found in both PGC1α and UCP1 mRNA expression in WAT, but not cultured EF, of GABA_B_R1-null mice. Gb1, GABA_B_R1; ND, not detectable.

To next investigate the possible significance other than the partner of functional GABA_B_R of GABA_B_R1 subunit expressed by adipocytic cells, we examined the expression profile of a variety of adipocyte marker genes in cultured EF prepared from GABA_B_R1-null mice. Similarly marked expression was seen with mRNA for a variety of adipocytic marker genes related to differentiation and lipolysis between EF from WT and GABA_B_R1-null mice (Figure 3A). These included PPARγ, CCAAT/enhancer binding protein-α (C/EBPα), Fabp4, LPL, β3 adrenergic receptor (AR) and perilipin. However, mRNA expression was significantly lower with hormone sensitive lipase (HSL) in EF of GABA_B_R1-null mice than that of WT mice. Among different adipocytokines examined, moreover, a significant decrease was seen in mRNA expression for leptin, but not for adiponectin, resistin or tumor necrosis factor-α (TNFα), in EF from GABA_B_R1-null mice. Independent of the presence of GABA_B_R1 subunit, by contrast, mRNA expression was not detected with the 2 marker genes related to mitochondrial biogenesis such as PGC1α and uncoupling protein 1 (UCP1) in EF cultured for 2 days. Transfection with GABA_B_R1 expression vector drastically increased GABA_B_R1 mRNA expression in EF cultured for 2 days (data not shown), but failed to prevent the reduction of leptin mRNA expression in EF prepared from GABA_B_R1-null mice (Figure 3B).

Among different marker genes examined, similarly, expression of leptin mRNA was selectively and significantly reduced in WAT isolated from GABA_B_R1-null mice compared with that of WT mice (Figure 3C). No significant changes were found in mRNA expression of PPARγ, C/EBPα, Fabp4, LPL, β3-AR, HSL and perilipin as adipocytic markers as well as adiponectin, resistin and TNFα as adipocytokines between WAT isolated from GABA_B_R1-null and WT mice. In contrast to cultured EF, however, marked mRNA expression was seen with mitochondrial biogenesis markers such as PGC1α and UCP1 in WAT irrespective of GABA_B_R1 subunit knockout. In WAT of GABA_B_R1-null mice, drastic upregulation was seen in mRNA expression of both PGC1α and UCP1 compared to WT mouse WAT. These results suggest that GABA_B_R1 subunit would play a role in adipogenesis through downregulation of leptin expression in a manner not related to functional GABA_B_R properties in adipocytes.

### Fat phenotypes in GABA_B_R1-null mice

**Figure 4:**
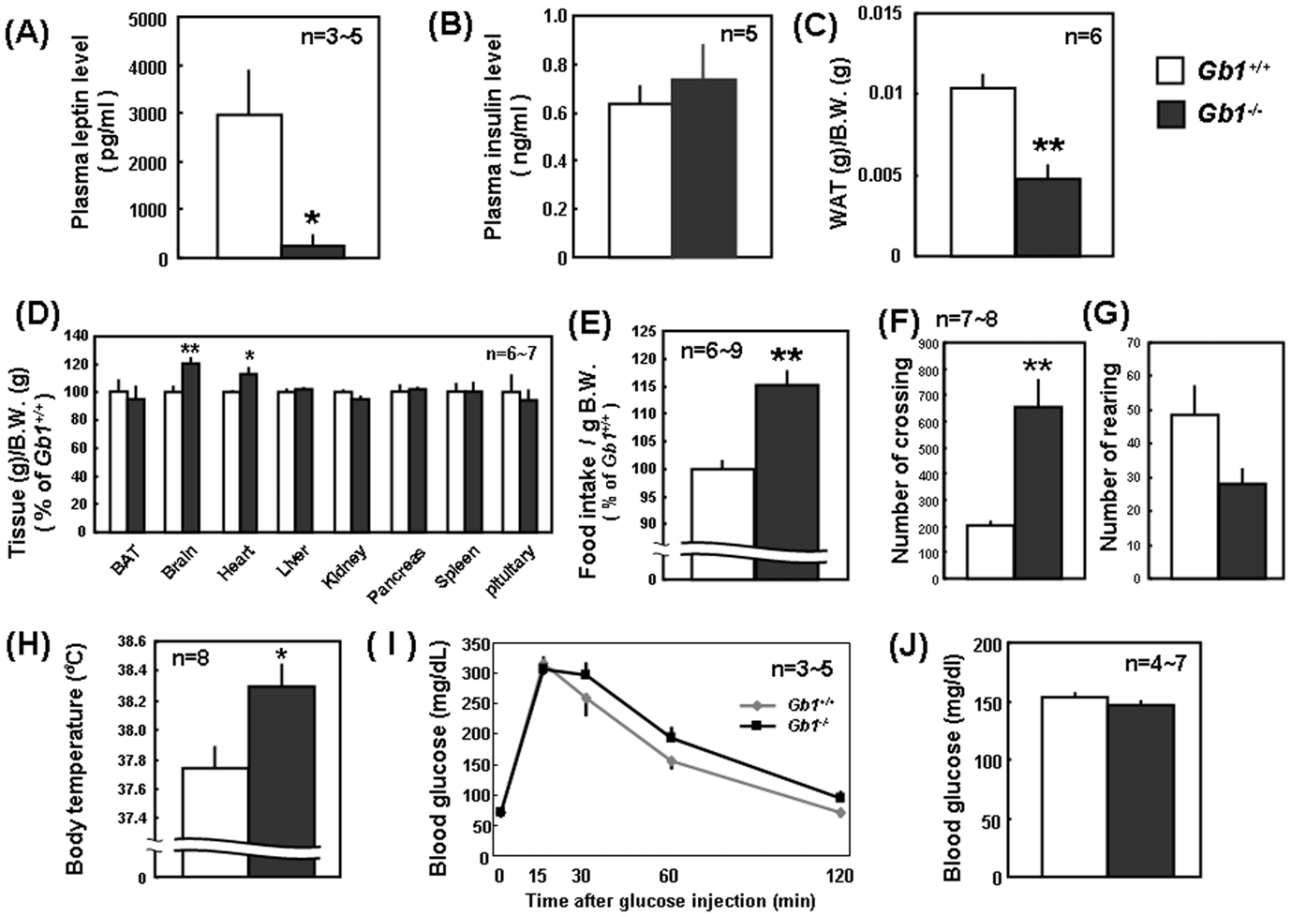
Phenotypes of GABA_B_R1-null mice. Plasma was collected from blood of male mice at 4 weeks old, followed by measurement of (A) leptin and (B) insulin using ELISA kits. Various tissues were dissected from male mice at 4 weeks old, followed by measurement of wet weights of (C) WAT and (D) other tissues for calculation of the percentage over body weight. (E) Mice were fed with powder diets for 1 week, followed by measurement of the accumulated amount of food intake during 1 week. Open-field tests were carried out using male mice at 4 weeks old for (F) the number of crossing and (G) the number of rearing. (H) Body temperature was measured at the rectum in mice. (I) Mice were fasted for 15 to 16 h and then injected ip with 2 g/kg glucose, followed by determination of blood glucose levels 15 to 120 min after the injection. (J) Basal blood glucose levels were measured in male mice at 4 weeks old. Values are the mean ± S.E. from different experiments shown in the figure. *P<0.05; **P<0.01, significantly different from each control value obtained in WT mice. B.W., body weight; Gb1, GABA_B_R1.

For further evaluation of the possible function of GABA_B_R1 subunit expressed by adipocytes, GABA_B_R1-null mice were analyzed for potential fat phenotypes under the normal chow diet at 4 weeks of age. GABA_B_R1-null mice exhibited a drastic decrease in plasma leptin levels (Figure 4A), with plasma insulin levels being unchanged (Figure 4B), compared with those in WT littermates. A significant reduction was seen in the ratio of WAT weight over body weight in GABA_B_R1-null mice compared with control littermates (Figure 4C), with no marked changes in the weights of other organs such as BAT, liver, kidney, pancreas, spleen and hypophysis (Figure 4D). By contrast, a significant increase was observed in the ratios of brain and heart weights over body weight in GABA_B_R1-null mice compared with those in WT mice.

To understand the reason for the lower body fat percentage in GABA_B_R1-null mice, food intake was monitored for 1 week in addition to spontaneous locomotor activity. Food intake was significantly increased in GABA_B_R1-null mice compared to WT littermates (Figure 4E), while horizontal movement (walking) was significantly increased in GABA_B_R1-null mice (Figure 4F) with no significant change in vertical activity (rearing) (Figure 4G). In addition, GABA_B_R1-null mice showed elevated core body temperature, which is an alternative measure of energy consumption, compared with WT littermates (Figure 4H). To assess the potential effects of GABA_B_R1 homozygosity on glucose homeostasis, glucose tolerance test was performed in GABA_B_R1-null mice. However, no significant changers were seen in glucose tolerance (Figure 4I) and plasma glucose levels (Figure 4J) in GABA_B_R1-null mice compared with controls. Accordingly, there seems to be a positive correlation between GABA_B_R1 subunit expression and plasma leptin levels.

### Transfection with GABA_B_R1 siRNA in 3T3-L1 cells

**Figure 5:**
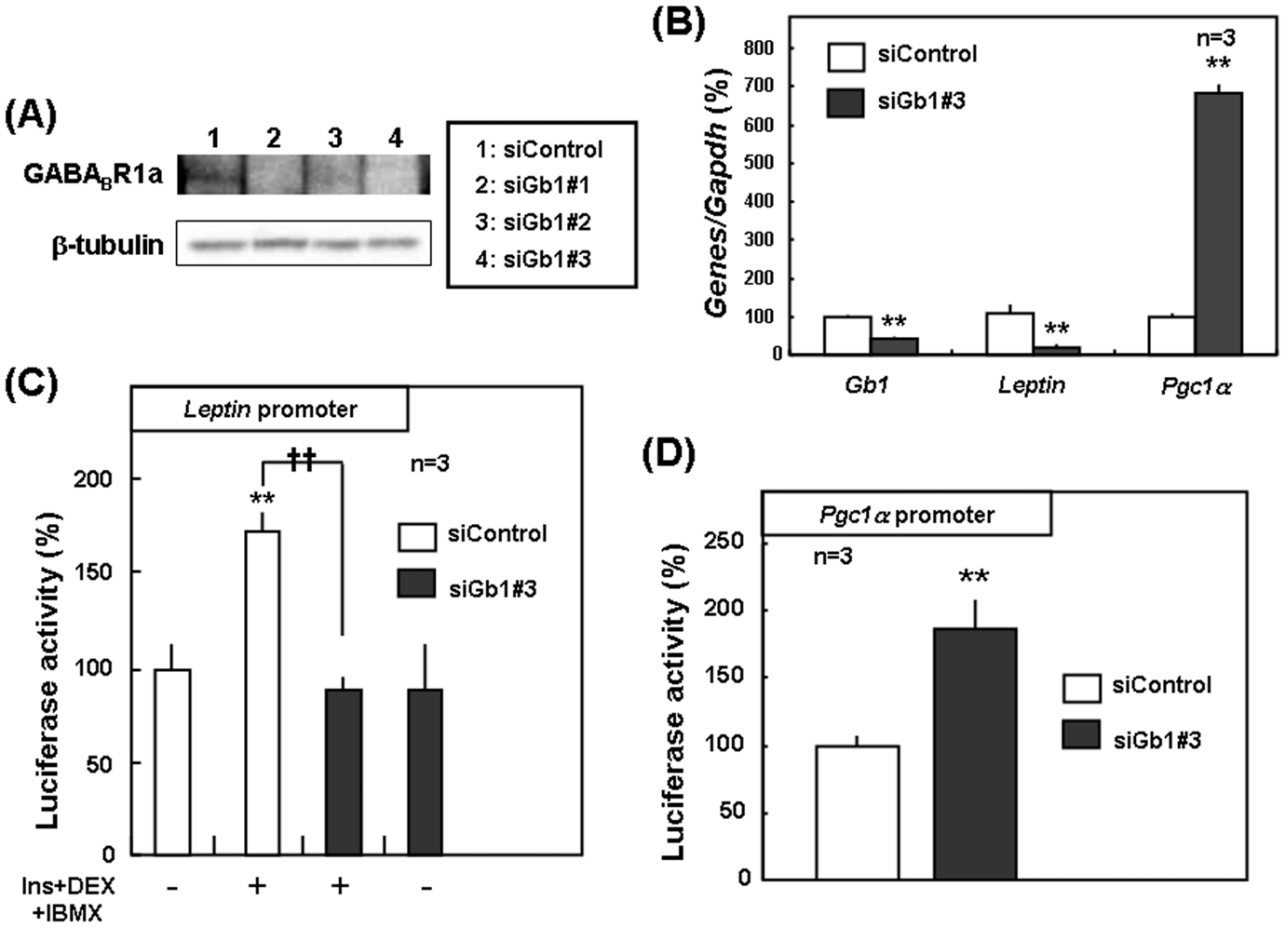
Transfection with GABA_B_R1 siRNA in 3T3-L1 cells. (A) 3T3-L1 cells transfected with si-control and si-GABA_B_R1 were cultured for 3 days, followed by immunoblotting for GABA_B_R1 subunit. (B) 3T3-L1 cells transfected with siRNA were cultured for 2 days in the presence of inducers of adipocytic differentiation, followed by real time-PCR for GABA_B_R1 subunit, leptin and PGC1α. (C) 3T3-L1 cells were transfected with the luciferase vector containing leptin promoter together with siRNA, followed by culture in either the presence or absence of inducers such as insulin, DEX and IBMX for 2 days and subsequent determination of luciferase activity. (D) 3T3-L1 cells were transfected with the luciferase vector containing PGC1α promoter along with siRNA, followed by culture for 3 days and subsequent determination of luciferase activity. Values are the mean ± S.E. from 3 different experiments. *P<0.05; **P<0.01, significantly different from each control value. ^††^P<0.01, significantly different from the value obtained in control cells cultured in the presence of inducers. Knockdown by siRNA of GABA_B_R1 subunit led to decreased leptin and increased PGC1α mRNA expression levels through modulation of transactivation of corresponding genes. Gb1, GABA_B_R1.

To evaluate a role of GABA_B_R1 subunit in leptin expression in adipocytes, 3T3-L1 cells were transiently transfected with siRNA for GABA_B_R1 subunit by RNA-mediated interference. GABA_B_R1 subunit protein levels were markedly decreased in 3T3-L1 cells transfected with the three different siRNA for GABA_B_R1 subunit for 72 h compared to cells with scrambled control siRNA (Figure 5A). In 3T3-L1 cells transfected with GABA_B_R1 siRNA, no marked alterations were found in fibroblastic morphology, viability and proliferation rate (data not shown). In cells with GABA_B_R1 siRNA#3, a significant decrease was seen in leptin mRNA expression with increased PGC1α mRNA expression (Figure 5B). To further explore the mechanisms underlying the regulation of leptin and PGC1α gene expression, luciferase reporter plasmids were constructed with leptin and PGC1α promoters, respectively, for subsequent transfection to 3T3-L1 cells. The addition of insulin, DEX and IBMX significantly increased luciferase activity with leptin promoter plasmid, while introduction of GABA_B_R1 siRNA#3 led to significant prevention of the increase in luciferase activity without significantly affecting basal leptin promoter activity (Figure 5C). By contrast, PGC1α promoter activity was significantly increased in 3T3-L1 cells transfected with GABA_B_R1 siRNA#3 for 72 h (Figure 5D). Therefore, GABA_B_R1 subunit protein would directly promote transactivation of leptin gene in adipocytic cells.

## Discussion

The essential importance of the present finding is that GABA_B_R1 subunit mRNA and protein were constitutively expressed by adipocytes to primarily regulate leptin expression at the transcriptional level. To our knowledge, this is the first direct demonstration of constitutive and functional expression of GABA_B_R1 subunit by adipocytes. In principle, the metabotropic GABA_B_R is orchestrated by a heterodimeric assembly of members of GABA_B_R1 and GABA_B_R2 subunits, neither of which is fully functional when individually expressed. Any GABA_B_R1 subunits are unable to activate voltage-sensitive K^+^ channels alone, whereas heterodimerization between GABA_B_R1a/1b and GABA_B_R2 subunits is required for the formation of fully functional GABA_B_R at the cell surface [20]–[23]. GABA_B_R1-deficient [24] and GABA_B_R2-deficient [25] mice have been developed and analyzed in the literature so far. Both of them show spontaneous seizures, hyperalgesia and severe memory impairment [24], [25], suggesting that heterodimerization between GABA_B_R1 and GABA_B_R2 subunits is critical for elicitation of most functions mediated by GABA_B_R. In GABA_B_R2-deficient mice, but not in GABA_B_R1-deficient mice, however, atypical electrophysiological GABA_B_ responses are still seen, suggesting that GABA_B_R1 subunit plays a functional role even in the absence of the dimerization partner, GABA_B_R2 subunit [25]. Indeed, we have previously demonstrated that GABA_B_R1, but not GABA_B_R2, subunit is expressed by osteoblasts with suppression of their differentiation by the GABA_B_R agonist baclofen [9], in contrast to the failures of a GABA_B_R agonist or antagonist to modulate cellular differentiation and forskolin-induced CRE reporter activity in 3T3-L1 cells in this study. Nevertheless, GABA_B_R1 subunit could interact with members other than GABA_B_R2 subunit in the G-protein-coupled receptor superfamily with seven transmembrane domains [26]. For instance, mGluR4 subunit has a high similarity to GABA_B_R with the ability for homodimerization [26]. Indeed, co-expression of GABA_B_R1a with mGluR4 leads to expression of GABA_B_R1a subunit at the cell surface, without formation of a heterodimer between GABA_B_R1a and mGluR4, or coupling to adenylyl cyclase and potassium channels [27]. Heterodimerization between kappa and delta opiate receptors as well as hetero-oligomerization between dopamine and somatostatin receptors occurs with functional activities different from those of either receptor in G-protein-coupled receptor superfamily with seven transmembrane domains as well [28], [29]. In any cases, the definitive conclusion should await the discovery and identification of the counterpart required for orchestration of the functional GABA_B_R through heterodimerization with one of the different GABA_B_R1 subunit expressed by adipocytes in future studies.

Leptin is an adipose tissue hormone that acts centrally to regulate multiple physiologic systems including those governing energy homeostasis, neuroendocrine axes and reproduction [30]–[34]. Leptin circulates in the blood and acts on the brain to regulate food intake. When fat mass falls, plasma leptin concentrations fall as well, stimulating appetite and suppressing energy expenditure until fat mass is restored. When fat mass increases, leptin levels increase, suppressing appetite until body weight is lost. This system maintains homeostatic control of adipose tissue mass [35]. However, the reason why the later introduction of GABA_B_R1 subunit failed to rescue the decreased leptin mRNA levels in EF from GABA_B_R1-null mice is not clarified so far. One possible but unproven speculation is that GABA_B_R1 expression vector could rescue impaired leptin mRNA expression upon introduction into matured adipocytes prepared from visceral fat of GABA_B_R1-null mice. In fact, leptin is shown to be highly expressed by matured, but not immature, adipocytes [30]. Accordingly, the possibility that marked reduction of plasma leptin levels would be at least in part derived from the events secondary to decreased fat mass in GABA_B_R1-null mice is not ruled out so far. From this point of view, it should be emphasized that transfection with GABA_B_R1 subunit siRNA led to drastically decreased leptin mRNA expression along with marked inhibition of leptin promoter activity elevated by adipocytic differentiation inducers in cultured 3T3-L1 cells. These data clearly give rise to an idea that GABA_B_R1 subunit, at least in part, directly promotes leptin mRNA expression through an unidentified mechanism relevant to leptin gene transactivation in adipocytic cells.

Although the regulatory mechanisms of leptin expression are not completely understood so far, it has been reported that several transcriptional factors are responsible for regulation of leptin gene expression. For instance, PPARγ negatively regulates the expression of leptin gene, with both C/EBP and specificity protein 1 positively regulating the leptin gene expression [36]–[38], whereas activating enhancer-binding protein 2β decreases the expression and secretion of leptin in adipocytic 3T3-L1 cells [39]. By contrast, GABA_B_R1 subunit is shown to directly interact with particular nuclear transcription factors, which are highly expressed by adipocytes [40], to modulate biosynthesis of inducible target proteins in the brain [41], [42]. The present findings that GABA_B_R1 siRNA drastically decreased leptin promoter activity in adipocytic 3T3-L1 cells and GABA_B_R1 deficiency led to marked reductions of leptin mRNA expression in both WAT and EF in culture, therefore, argue in favor of an idea that GABA_B_R1 subunit could positively regulate leptin promoter activity at the transcription level as a transcriptional co-effector in adipocytes. The exact mechanism underlying the aforementioned interaction of GABA_B_R1 subunit with particular transcription factors on leptin expression, however, remains to be elucidated in future studies.

It is noteworthy that GABA_B_R1 siRNA drastically increased PGC1α expression at the transcriptional level in 3T3-L1 cells. PGC1α is initially identified as a cold-inducible coactivator for PPARγ in brown fat, whereas subsequent studies reveal that PGC1α is able to bind to many nuclear receptors and several other transcription factors outside the nuclear receptor superfamily to facilitate their transcriptional activities [43]. Therefore, PGC1α would play an important role in the mechanism associated with energy metabolism changes in response to external stimuli in different tissues. Overexpression of PGC1α is shown to activate mitochondrial biogenesis and cellular respiration in cultured murine cardiac myocytes [44], transgenic mouse muscles [45] and cultured myoblasts [46]. Consistent with a regulatory role in the cellular adaptation to increased energy requirements, the expression of PGC1α is highly responsive to nutritional and environmental stimuli. For example, PGC1α mRNA is strongly induced in BAT by cold exposure and in skeletal muscle following bouts of physical activity [43], [46], [47]. Increased PGC1α levels lead to enhanced mitochondrial electron transport activities that enable cells to meet rising energy demands during adaptive thermogenesis in BAT and contraction in muscle. The present paradoxical phenotypes between adipose tissue mass and food intake could be at least in part accounted for by taking into consideration the possible higher energy consumptions due to increased PGC1α expression in WAT of GABA_B_R1-null mice than of WT mice. In addition to a crucial role as a dimerization partner for the negative autocrine regulation mediated by GABA_B_R of insulin secretion in pancreatic β-cells [13], GABA_B_R1 subunit would be also involved in the expression of endocrine molecule leptin by adipocytes with the critical importance as a regulator of energy homeostasis in mammals.

It thus appears that GABA_B_R1 subunit is constitutively expressed by adipocytes to promote gene expression of the adipocytic hormone leptin through a mechanism not relevant to the heterodimeric assembly to functional GABA_B_R with GABA_B_R2 subunit. GABA_B_R1 subunit is thus a potential target for the discovery and development of an innovative drug useful for the therapy and treatment of a variety of lifestyle related diseases with accumulated adipose in human beings.
